# Bioelectrical Impedance Analysis and Manual Measurements of Neck Circumference Are Interchangeable, and Declining Neck Circumference Is Related to Presarcopenia

**DOI:** 10.1155/2021/6622398

**Published:** 2021-03-29

**Authors:** Masaaki Machino, Kei Ando, Kazuyoshi Kobayashi, Hiroaki Nakashima, Satoshi Tanaka, Shunsuke Kanbara, Sadayuki Ito, Taro Inoue, Hiroyuki Koshimizu, Taisuke Seki, Shinya Ishizuka, Yasuhiko Takegami, Yukiharu Hasegawa, Shiro Imagama

**Affiliations:** ^1^Department of Orthopedic Surgery, Nagoya University Graduate School of Medicine, Japan; ^2^Department of Orthopedic Surgery, Konan Kosei Hospital, Japan; ^3^Department of Rehabilitation, Kansai University of Welfare Sciences, Japan

## Abstract

**Purpose:**

Preventive medicine is important in an aging society. Presarcopenia is the preliminary stage of sarcopenia. Recent advances in bioelectrical impedance analysis (BIA) devices have enabled automatic estimation of neck circumference (NC). However, the agreement between and interchangeability of NC measured manually and that calculated with BIA have not been evaluated. We performed these analyses in the context of health checkups and investigated their associations with presarcopenia.

**Methods:**

We enrolled 318 participants who underwent anthropometric measurements, including NC measured manually and by BIA; assessment of physical function; and blood testing. We used Bland-Altman analysis to calculate the agreement between and interchangeability of NC measurements by BIA and by the manual method. We then statistically compared normal participants and those with presarcopenia. Using multivariable analysis, we subsequently investigated significant risk factors for presarcopenia. We defined presarcopenia according to the appendicular skeletal muscle index (aSMI; the ratio of arm and leg skeletal muscle mass to height^2^).

**Results:**

Bland-Altman analysis showed that bias (BIA-manual) was negative overall (−1.07), for male participants (−1.23), and for female participants (−0.96). This finding suggests that BIA measurement is an underestimate in comparison with manual measurement. NC measurement by BIA was found to be interchangeable with that by manual methods, inasmuch as the percentage error was less than 5% overall (4.38%), for male participants (3.81%), and for female participants (4.58%). Univariable analysis revealed that NC was significantly smaller in the participants with presarcopenia than in those without. Multivariable analysis, adjusted for confounding factors, revealed that a decrease in NC was significantly correlated with presarcopenia.

**Conclusions:**

BIA measurements of NC are interchangeable within about 95% with manual measurements. The decrease in NC measured by BIA was significantly associated with presarcopenia in both genders. NC measurement can be used for early detection of presarcopenia.

## 1. Introduction

Sarcopenia, a syndrome characterized by progressive and generalized loss of skeletal muscle mass and strength, is associated with adverse outcomes such as physical disability, poor quality-of-life (QOL), and death [1, 2]. To measure muscle mass, the Asia Working Group for Sarcopenia (AWGS) recommended using the skeletal muscle index, defined by the ratio of appendicular skeletal muscle mass to height squared [3]. The European Working Group on Sarcopenia in Older People (EWGSOP) defined low muscle mass only as “presarcopenia” [4].

Presarcopenia is the preliminary stage of sarcopenia. To establish better public health policy and devise prevention strategies, the prevalence and temporal trends of presarcopenia and related body composition measurements must be understood in relation to sex, age, and race. Evidence-based prevention strategies for sarcopenia and musculoskeletal diseases must be based on the incidence of disability, risk factors, and other epidemiological data [5]. Few such strategies have been developed, however, and the risk factors for presarcopenia that are associated with anthropometric markers in middle-aged and elderly people remain unclear [6, 7].

Neck circumference (NC), an anthropometric marker for detecting risk for metabolic disorders [8, 9], can also reflect upper body fat deposition and thereby help identify individuals at high risk for these disorders [10]. Increased NC can reflect elevated blood pressure, insulin resistance, lipid abnormalities, and the presence of metabolic syndrome [11].

Bioelectrical impedance analysis (BIA), a method of easily measuring water content and body fat mass, is commonly performed during general medical examinations [12, 13]. Recent advances in BIA devices have enabled clinicians to quickly estimate not only NC but also the circumferences of the chest, abdomen, and hips [14]. Nevertheless, BIA and manual measurements of NC have not been validated, particularly with regard to interrater reliability, agreement, and interchangeability.

Therefore, the purpose of this study was to validate the measurement of NC by both BIA and manual methods and to evaluate whether these two measurements are interchangeable. We also investigated whether NC measurement by BIA is correlated with presarcopenia according to gender during general health checkups in a large, prospective population.

## 2. Materials and Methods

### 2.1. Participants

The study participants were volunteers who underwent health checkups supported by the local government of Yakumo, Japan, in 2019. Of Yakumo's population of approximately 17,000 people, 28% are older than 65 years. A sizable proportion of its residents are engaged in agriculture and fishery. Since 1982, health checkups have been conducted annually in Yakumo; they consist of voluntary orthopedic and physical function examinations, internal medical examinations, and psychological tests, and a health-related QOL survey is administered as well [15–19]. In our participants, we obtained blood samples; conducted anthropometric measurements, including manual and BIA measurements of NC; and assessed physical function, in that order. On the basis of previous reports, participants underwent these evaluations after overnight fasting [20].

We excluded from this study subjects with neck masses (e.g., goiter or cervical lymphadenopathy), thyroid diseases, a history of spine and limb joint surgery, severe knee injury, severe osteoarthritis, a history of hip or spine fracture, neurological disorders, severe mental illness, diabetes, kidney or heart disease, and severe disability in walking or standing or any dysfunction of the central or peripheral nervous system. We also excluded data from participants who did not fast before testing. In addition, we excluded patients with sarcopenia (defined as reduced muscle mass and either reduced muscle strength or performance) and severe sarcopenia (defined as reduced muscle mass, reduced strength, and reduced performance) in order to focus on presarcopenia. Of the 537 individuals who underwent the health checkups, 318 participants (125 men and 193 women) met the selection criteria.

The study protocol was approved by the ethics committee of human research and the institutional review board of our university (approval no. 2014-0207). All participants provided written informed consent to participate before the study. The study procedures were carried out in accordance with the principles of the Declaration of Helsinki.

### 2.2. Anthropometric Measurements

Through BIA, we collected anthropometric data: weight, body mass index (BMI), percentage of body fat (PBF), appendicular skeletal muscle index (aSMI) to represent muscle mass, and NC. We used the InBody 770 body composition and body water analyzer (InBody, Seoul, Republic of Korea), a BIA unit, to differentiate tissues (such as fat, muscle, and bone) according to their electrical impedance [12, 13]. Measurements by this device in various contexts have been reported to be accurate [21, 22]. Electrodes are embedded in the handles of the analyzer, which were grasped by each participant, and in the platform, on which the soles of the participant's feet rested; two electrodes were in contact with each foot and hand. BMI was calculated as body weight (in kilograms) divided by body height squared (in meters). PBF was calculated as fat mass (in kilograms) divided by body weight (in kilograms) × 100. The aSMI was calculated as arm and leg skeletal muscle mass (in kilograms) divided by body height squared (in meters) [23]. NC was calculated twice automatically by the InBody 770 BIA device, and mean data were adopted [13].

### 2.3. Definition and Diagnosis of Presarcopenia

Presarcopenia is characterized by a reduction in muscle mass that does not affect muscle strength or physical performance, according to the EWGSOP [1]. The cutoff aSMI values (7.0 kg/m^2^ for men and 5.7 kg/m^2^ for women, calculated with BIA) were based on the diagnostic criteria described by the AWGS [3, 24].

### 2.4. Manual Measurements of Neck Circumference

While the participants stood with the head positioned in the Frankfort horizontal plane and the shoulders relaxed, we used a nonstretchable plastic tape to manually measure NC from the level just below the laryngeal prominence perpendicular to the long axis of the neck; measurements were performed twice by two independent investigators, and mean data were recorded in centimeters and rounded to the nearest millimeter [25–27]. Intraobserver and interobserver variations regarding the measurement of NC by manual methods were confirmed using the reliability statistics by intraclass correlation coefficient (ICC) by two independent observers, and the mean intra- and interobserver ICCs were 0.94 and 0.92, respectively. As a result, this measurement was considered to be reasonable.

### 2.5. Physical Performance

To measure grip strength, we used the Toei Light Handgrip Dynamometer (Toei Light Co., Saitama, Japan) [6, 28]. Participants were in the standing position, and both hands were tested once; the average value was used as the participant's grip strength. To measure back muscle strength (the maximal isometric strength of the trunk muscles), we used a digital back muscle strength meter (T.K.K.5402; Takei Scientific Instruments Co., Niigata, Japan) while participants were in a standing position in 30° of lumbar flexion [13, 29]. To evaluate mobility, participants performed two tasks: (1) they walked a straight 10 m course one time at their fastest pace, and the time necessary to complete the course was recorded as the 10 m gait time [6, 28], and (2) they rose from a standard chair (46 cm seat height from the ground), walked a distance of 3 m, turned around, walked back to the chair, and sat down (the 3 m timed up-and-go test (3-m TUG)), and the time necessary to accomplish this was measured twice, and the mean of the two measurements was recorded [13, 29].

### 2.6. Blood Tests

We analyzed venous blood samples from each participant for levels of albumin (a marker of nutritional status), total cholesterol and triglycerides (which can indicate the presence of metabolic syndrome), and C-reactive protein (a marker of inflammation). Biochemical analyses of the blood samples were performed with the use of an autoanalyzer (JCA-RX20; Nihon Denshi, Tokyo, Japan) [12, 13].

### 2.7. Statistical Analysis

All data were analyzed with SPSS statistical software (version 25.0; SPSS Statistics, IBM Corp., Armonk, NY, USA). We calculated continuous variables as means and standard deviations (SDs) and categorical variables as percentages. We used the Mann–Whitney *U* test and the chi-square test to evaluate between-group differences, as appropriate for the data distribution. We examined correlations between manual and BIA measurements of NC by using the Spearman *r* and ICC (absolute agreement, two-way random, and single measures). To interpret Spearman correlations, cutoff values of less than 0.20 were considered very weak; 0.20 to 0.39, as weak; 0.40 to 0.59, as moderate; 0.60 to 0.79, as strong; and 0.80 to 1.0, as very strong [26, 30]. To interpret the ICC, cutoff values of less than 0.20 were considered slight; 0.20 to 0.39, as fair; 0.40 to 0.59, as moderate; 0.60 to 0.79, as substantial; and 0.80 to 1.0, as almost perfect [12]. To examine the level of agreement between the manual and BIA measurements, we used the Bland-Altman analysis [12]. The mean of the difference between measurements (BIA versus manual) was defined as bias, and SD was also used to calculate 95% confidence limits of agreement (bias: ±1.96 SD). The Bland-Altman plots graphically displayed the mean of the two measured values (NC measurement with BIA and the manual method) on the *x*-axis and the difference (BIA versus manual) between measured values on the *y*-axis. To determine whether BIA measurements were interchangeable with manual measurements, we used a percentage error (the ratio of 1.96 SD to the mean value of the manual method) as 8% or less [31, 32]. To determine the factors associated with presarcopenia among the variables that exhibited differences (*p* < 0.01) in the univariable analyses, logistic regression analysis using a stepwise method was performed using the aforementioned variables as covariables. A *p* value of < 0.05 was considered significant in all analyses.

## 3. Results

The average age of the 318 participants was 63.4 years (range, 40–87 years; SD, 10.0 years), the average BMI was 23.7 kg/m^2^, and the average PBF was 29.4%.  Table 1 lists the demographic data, anthropometric measurements, physical function results, blood test results, and presarcopenia prevalence. Men and women exhibited significant differences for all variables except blood test data. Both manual and BIA measurements of NC were smaller in women than in men. The prevalence of presarcopenia was higher in women than in men.

Table 2 lists Spearman *r*, ICC, and Bland-Altman analysis results for the NCs as measured by the two methods. BIA measurements were found to be interchangeable with manual measurements, inasmuch as the percentage error was less than 5% (4.38% overall, 3.81% for men, and 4.58% for women). The Bland-Altman plots showed that BIA measurements of NC were in almost perfect agreement with manual measurements, according to ICC (Table 2).

**Figure 1:**
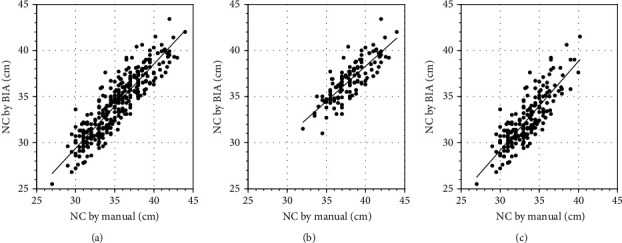
Scatterplot of neck circumference (NC) by bioelectrical impedance analysis (BIA) and by the manual method. Measurement of NC by BIA was significantly and very strongly positively correlated with that by the manual method. (a) Total (*r* = 0.90; *p* < 0.0001), (b) in male participants (*r* = 0.81; *p* < 0.0001), and (c) in female participants (*r* = 0.83; *p* < 0.0001).

**Figure 2:**
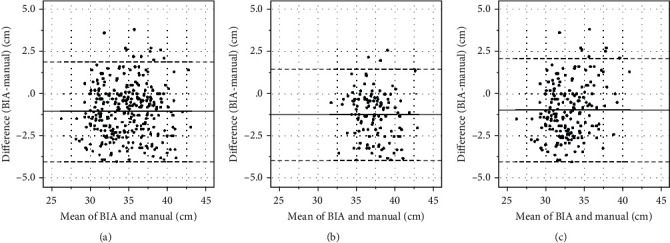
Bland-Altman plot of difference in neck circumference (NC)—measurement with bioelectrical impedance analysis minus manual measurement—against the mean of two measurements. The middle line denotes bias (mean difference between the two measurements), and the dashed lines denote 95% limits of agreement (1.96 standard deviation from the difference). (a) Total (bias: −1.07; 95% limits of agreement (LOA): −4.02 to 1.89), (b) in male participants (bias: −1.23; 95% LOA: −3.96 to 1.50), and (c) in female participants (bias: −0.96; 95% LOA: −4.04 to 2.12).

BIA measurements of NC were very strongly correlated manual measurements of NC overall (Spearman *r* = 0.90, *p* < 0.0001), for men (Spearman *r* = 0.81, *p* < 0.0001), and for women (Spearman *r* = 0.83, *p* < 0.0001; Figure 1). The Bland-Altman analysis showed that bias (BIA versus manual) was negative overall (−1.07), for men (−1.23), and for women (−0.96), which suggested that BIA measurements were underestimates in comparison with manual measurements (Figure 2).

Table 3 lists the results of the comparisons between the normal participants and those with presarcopenia, by gender. Overall, all variables differed significantly between the two groups, except for age and blood test data. Both men and women with presarcopenia had lower body weights and smaller NCs according to manual and BIA measurements than did the normal participants. The physical function of both men and women with presarcopenia was inferior to that of the normal men and women (Table 3).

The results of the logistic regression model for presarcopenia in all participants are listed in Table 4. BIA measurement of NC (*p* < 0.001), body weight (*p* < 0.001), grip strength (*p* < 0.001), back muscle strength (*p* < 0.001), PBF (*p* = 0.003), and female gender (*p* = 0.025) were significantly associated with presarcopenia. The results of the logistic regression model according to sex are listed in Table 5. BIA measurement of NC was significantly associated with presarcopenia in both men and women.

**Figure 3:**
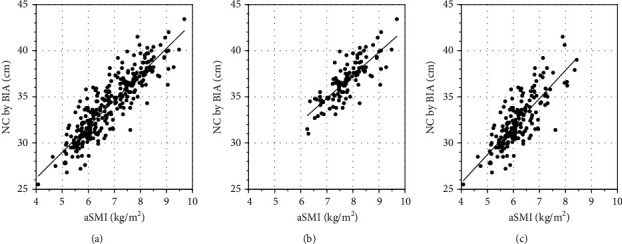
Scatterplot of neck circumference (NC) by bioelectrical impedance analysis (BIA) and by appendicular skeletal muscle index (aSMI). Measurement of NC by BIA was significantly and very strongly positively correlated with that by aSMI. (a) Total (*r* = 0.89; *p* < 0.0001), (b) in male participants (*r* = 0.80; *p* < 0.0001), and (c) in female participants (*r* = 0.80; *p* < 0.0001).

BIA measurement of NC was significantly and very strongly positively correlated with aSMI overall (*r* = 0.89, *p* < 0.0001), for men (*r* = 0.80, *p* < 0.0001), and for women (*r* = 0.80, *p* < 0.0001; Figure 3). From these results, we found that the decline in BIA measurements of NC was significantly associated with presarcopenia, which indicates a decrease in aSMI.

## 4. Discussion

Sarcopenia is an important barometer of disability and frailty in elderly people, inasmuch as it exacerbates poor general health or frailty [2, 5]. Presarcopenia is the preliminary stage of sarcopenia [6]. The causes of sarcopenia are varied and complex; they include disuse of muscles as a result of malnutrition, vitamin D deficiency, cerebral infarction, heart failure, and osteoarthritis; age-related changes in levels of hormones such as testosterone, estrogen, insulin-like growth factor 1, and insulin; apoptosis, denervation, inflammation, and changes in immunity involving interleukin- (IL-) 1, IL-6, and tumor necrosis factor-*α*; and social and mental causes, such as decline in cognitive function or decreased social activity [33–37]. Loss of appendicular skeletal muscle mass is a predictor of mortality in elderly people [3, 38]. Intervention at the presarcopenia stage is necessary to prevent sarcopenia. Therefore, indicators of presarcopenia must be established.

Interest in NC as a clinical measurement has increased substantially [26]. Unlike waist circumference, the measurement of which differs widely in location, and the location of NC measurement is standard and straightforward. Meals, respiration, and position have no effect on NC measurement [25]. NC can be measured without the need to remove clothing and can also be measured in pregnant and ascitic patients [26].

In general, NC measurement is performed manually. In large-scale health checkups, manual measurements of many patients within a limited time may lead to interrater error because of the increasing numbers of clinicians who perform the measurements. To solve such problems, a device that can be used to perform automatic measurements in a short time must yield measurements that correspond to manual measurements. Portable BIA devices have been used to measure body composition and anthropometric markers [20]. Clinicians also use them in the context of health checkups. Because BIA can account for a large amount of data from one measurement in a short period of time, the labor, time, and interrater errors involved are reduced in comparison with those associated with manual measurement [12]. As a result of technological advances, BIA devices that can automatically measure anatomical circumferences, including NC, have been developed [13]. Nevertheless, BIA has not been validated with regard to manual measurement values. Our study was the first in which these two methods of measuring NC were validated in a large-scale prospective population of patients undergoing health checkups.

Moreover, BIA devices have been used to measure PBF and aSMI in various studies, and so we hypothesized that additional evidence could be accumulated [21, 22]. aSMI is a well-accepted measure for the screening of presarcopenia [6]. The use of BIA as a noninvasive and easy-to-use method for evaluation of sarcopenia has increased [2]. Our results also demonstrated that BIA measurement of NC was independently associated with aSMI.

With regard to accuracy, BIA and manual measurements of NC must be in agreement because manual measurement can introduce errors. If the agreement is high, it is possible that the measurement modalities are interchangeable. In general, Spearman correlations and ICC are used to validate the values obtained with the two measurement methods. However, correlation analysis concerns the relationship between two different events, and the value of the correlation coefficient cannot reveal differences between or variations in measurements [12]. Therefore, these assessments alone are insufficient for evaluating interchangeability. The Bland-Altman analysis, a method of measuring two methods and examining the differences between the measurements, is also necessary. We evaluated the interchangeability of BIA and manual measurements on the basis of past reports [2, 12]. For men and women, BIA and manual measurements of NC were strongly correlated and thus demonstrated the interchangeability of the two methods. This result is very important, and in future research, investigators can focus on the use of BIA not only for NC but also for other anatomical circumferences.

Furthermore, because we confirmed that BIA measurement of NC is interchangeable with manual measurement, we examined the results of a previous report in which NC was not measured with BIA and presarcopenia as the preliminary stage of sarcopenia was not considered [13]. The univariable analysis finding in this study was that BIA measurements of NC were significantly smaller in participants with the presarcopenia group overall and by gender. Moreover, even if statistical adjustments were made for other factors in logistic regression analysis, a decline in NC as measured with BIA was a significant risk factor for presarcopenia. This study allowed us to build upon the previous report and accumulate further evidence. Declining NC will serve as a predictive factor for presarcopenia in the future, and it may be used to evaluate therapeutic effect. The results of this study indicate that additional research on sarcopenia is warranted.

This study had some limitations. First, data from only one race of people in a single center were analyzed, and their demographic characteristics did not reflect those of people dwelling in the general community. The subjects were healthy middle-aged and elderly people who lived in a relatively rural area, and many had jobs in agriculture or fishing; therefore, they did not represent people living in an urban environment [28, 29]. Second, this study was a cross-sectional study. A longitudinal larger-scale study is necessary to identify the causes of presarcopenia. Third, BIA devices from different manufacturers may yield different measurements. Therefore, standardization of technology and cross-calibration of electrical resistance should be addressed. It is necessary to further increase the number of participants and verify the justification in future studies.

The progress of BIA technology has been remarkable; depending on the device, it is possible to measure NC, and a large amount of data can be obtained in a short time from one measurement. The results of this study indicate that the relationship between NC and various diseases and conditions might be investigated on a large scale. We hope that the simple measurement of NC will play an important role in screening for presarcopenia and that early intervention in the form of muscle-strengthening exercises may prevent the conversion to sarcopenia.

## 5. Conclusion

BIA measurements of NC are interchangeable within about 95% with manual measurements. The decline in NC as measured by BIA was significantly associated with presarcopenia in both genders. NC measurement may play a role in the early detection of presarcopenia.

## Figures and Tables

**Table 1:** Demographic, anthropometric, physical function, blood test, and presarcopenia prevalence data of the study participants.

Variables	Total	Male	Female	*p*
Number of subjects	318	125	193	
Age (years)	63.4 ± 10.0	66.0 ± 9.19	61.8 ± 10.2	**0.0002**
Body height (cm)	158.8 ± 8.45	165.6 ± 6.66	154.4 ± 6.23	**<0.0001**
Body weight (kg)	60.2 ± 11.7	67.3 ± 9.93	55.5 ± 10.4	**<0.0001**
BMI (kg/m^2^)	23.7 ± 3.63	24.5 ± 2.92	23.3 ± 3.95	**0.0017**
PBF (%)	29.4 ± 7.54	23.8 ± 4.24	33.0 ± 6.98	**<0.0001**
aSMI (kg/m^2^)	6.82 ± 1.05	7.76 ± 0.71	6.19 ± 0.71	**<0.0001**
NC by BIA (cm)	34.1 ± 3.32	36.8 ± 2.19	32.4 ± 2.78	**<0.0001**
NC by manual (cm)	35.2 ± 3.26	38.0 ± 2.34	33.4 ± 2.38	**<0.0001**
Grip strength (kg)	28.8 ± 9.03	37.7 ± 6.62	23.2 ± 4.84	**<.0001**
Back muscle strength (kg)	83.6 ± 51.2	112.9 ± 26.1	63.8 ± 54.5	**<0.0001**
10 m gait time (s)	5.00 ± 0.89	4.76 ± 0.79	5.17 ± 0.93	**0.0001**
TUG (s)	5.98 ± 1.06	5.74 ± 1.02	6.15 ± 1.05	**0.0014**
Albumin (g/dL)	4.36 ± 0.24	4.37 ± 0.27	4.36 ± 0.21	0.6785
Total cholesterol (mg/dL)	206.9 ± 34.0	195.9 ± 33.3	214.0 ± 32.5	**<0.0001**
Triglycerides (mg/dL)	92.9 ± 55.2	98.6 ± 54.7	89.2 ± 55.5	0.1492
CRP (mg/dL)	0.11 ± 0.35	0.16 ± 0.49	0.08 ± 0.21	0.1266
Prevalence of presarcopenia (%)	60, 18.9%	16, 12.8%	44, 22.8%	**0.0357**

Evaluated using the Mann–Whitney *U* test and chi-square test. Parameter values are shown as means (standard deviations) or numbers. Bold values indicate significant difference. PBF, aSMI, and NC by BIA were measured using InBody 770 BIA unit. BMI: body mass index; PBF: percent body fat; aSMI: appendicular skeletal muscle index; NC: neck circumference; BIA: bioelectrical impedance analysis; TUG: timed up-and-go; CRP: C-reactive protein.

**Table 2:** Correlation coefficient (*r*), ICC, and Bland-Altman analysis in NC measured by two methods: manual and BIA.

	Total	Male	Female
Spearman *r*	0.90^∗∗∗^	0.81^∗∗∗^	0.83^∗∗∗^
ICC	0.95	0.90	0.90
Bland-Altman analysis			
Bias (BIA-manual)	−1.07	−1.23	−0.96
SD	1.51	1.39	1.57
95% LOA	−4.02 to 1.89	−3.96 to 1.50	−4.04 to 2.12
Percentage error (%)	4.38	3.81	4.58

^∗∗∗^ *p* < 0.0001; *r*: correlation coefficient; ICC: interclass correlation coefficients; NC: neck circumference; BIA: bioelectrical impedance analysis; SD: standard deviation; LOA: limits of agreement.

**Table 3:** Comparison between the normal group and the presarcopenia group according to sex.

Variables	Total			Male			Female		
	Normal	Presarcopenia	*p*	Normal	Presarcopenia	*p*	Normal	Presarcopenia	*p*
Number of subjects	257	60		109	16		148	44	
Age (years)	63.0 ± 9.98	65.2 ± 10.0	0.1261	65.6 ± 9.33	68.7 ± 7.88	0.2142	61.1 ± 10.0	63.9 ± 10.5	0.1067
Sex (male/female)	109/148	16/44	**0.0357**						
Body height (cm)	159.7 ± 8.32	155.2 ± 8.00	**0.0003**	165.9 ± 6.68	164.1 ± 6.52	0.3235	155.2 ± 6.23	151.8 ± 5.48	**0.0019**
Body weight (kg)	62.8 ± 11.0	48.9 ± 6.71	**<0.0001**	69.1 ± 9.28	55.0 ± 3.33	**<0.0001**	58.2 ± 9.83	46.6 ± 6.22	**<0.0001**
BMI (kg/m^2^)	24.5 ± 3.40	20.4 ± 2.41	**<0.0001**	25.1 ± 2.59	20.5 ± 1.54	**<0.0001**	24.1 ± 3.85	20.3 ± 2.68	**<0.0001**
PBF (%)	30.0 ± 7.59	26.6 ± 6.76	**0.0026**	24.4 ± 4.00	20.0 ± 3.75	**<0.0001**	34.1 ± 6.96	29.4 ± 5.73	**0.0002**
aSMI (kg/m^2^)	7.07 ± 0.96	5.75 ± 0.65	**<0.0001**	7.93 ± 0.61	6.68 ± 0.17	**<0.0001**	6.43 ± 0.61	5.38 ± 0.31	**<0.0001**
NC by BIA (cm)	34.9 ± 3.07	31.1 ± 2.41	**<0.0001**	37.2 ± 2.01	34.0 ± 1.13	**<0.0001**	33.2 ± 2.59	30.0 ± 1.78	**<0.0001**
NC by manual (cm)	35.8 ± 3.14	32.6 ± 2.31	**<0.0001**	38.4 ± 2.18	35.2 ± 1.17	**<.0001**	33.9 ± 2.27	31.7 ± 1.88	**<0.0001**
Grip strength (kg)	30.0 ± 9.13	24.1 ± 6.69	**<0.0001**	38.5 ± 6.56	32.6 ± 4.58	**0.0005**	23.9 ± 4.86	20.8 ± 3.73	**0.0001**
Back muscle strength (kg)	88.5 ± 54.5	62.4 ± 23.5	**<0.0001**	116.0 ± 25.7	91.6 ± 18.0	**0.0010**	67.7 ± 60.9	50.7 ± 12.8	**0.0004**
10 m gait time (s)	4.94 ± 0.88	5.25 ± 0.84	**0.0126**	4.72 ± 0.74	4.97 ± 1.05	0.5545	5.10 ± 0.94	5.37 ± 0.72	**0.0279**
TUG (s)	5.91 ± 1.06	6.24 ± 0.98	**0.0373**	5.67 ± 1.00	6.15 ± 1.13	0.0857	6.10 ± 1.08	6.27 ± 0.92	0.4030
Albumin (g/dL)	4.37 ± 0.24	4.33 ± 0.22	0.2497	4.37 ± 0.27	4.33 ± 0.23	0.4724	4.36 ± 0.21	4.33 ± 0.22	0.3842
Total cholesterol (mg/dL)	206.9 ± 34.0	206.7 ± 34.3	0.7757	197.3 ± 32.6	187.1 ± 37.7	0.3766	213.9 ± 33.4	214.5 ± 30.0	0.6965
Triglycerides (mg/dL)	96.1 ± 58.6	78.9 ± 34.5	0.0705	101.4 ± 56.9	80.6 ± 33.5	0.2893	92.3 ± 59.8	78.3 ± 35.4	0.2184
CRP (mg/dL)	0.11 ± 0.27	0.14 ± 0.59	0.1521	0.13 ± 0.30	0.36 ± 1.10	0.6940	0.09 ± 0.24	0.06 ± 0.08	0.2622

Evaluated using the Mann–Whitney *U* test and chi-square test. Parameter values are shown as means (standard deviations). Bold values indicate significant difference. BMI: body mass index; PBF: percent body fat; aSMI: appendicular skeletal muscle index; NC: neck circumference; BIA: bioelectrical impedance analysis; TUG: timed up-and-go; CRP: C-reactive protein.

**Table 4:** Logistic regression model for presarcopenia in all the participants.

Variables	*β*	Odds ratio (95% CI)	*p*
NC by BIA (cm)	-0.49	0.61 (0.53-0.70)	**<0.001**
Body weight (kg)	-0.19	0.82 (0.78-0.87)	**<0.001**
Grip strength (kg)	-0.09	0.91 (0.87-0.95)	**<0.001**
Back muscle strength (kg)	-0.03	0.97 (0.96-0.99)	**<0.001**
PBF (%)	-0.07	0.94 (0.90-0.98)	**0.003**
Sex (male)	-0.71	0.49 (0.26-0.91)	**0.025**
Age (years)			0.116

All variables (*p* < 0.01) that showed a certain degree of difference in univariate analysis were used as covariates. The dependent variable was presarcopenia. Covariates were age, sex, body weight, PBF, NC by BIA, grip strength, and back muscle strength. Bold values indicate significant difference. *β*: partial regression coefficient; CI: confidence intervals; NC: neck circumference; BIA: bioelectrical impedance analysis; PBF: percent body fat.

**Table 5:** Logistic regression model for presarcopenia according to sex.

Variables	Male			Female		
	*β*	Odds ratio (95% CI)	*p*	*β*	Odds ratio (95% CI)	*p*
NC by BIA (cm)	-1.18	0.31 (0.17-0.54)	**<0.001**	-0.80	0.45 (0.34-0.59)	**<0.001**
Body weight (kg)	-0.34	0.71 (0.61-0.83)	**<0.001**	-0.25	0.78 (0.72-0.85)	**<0.001**
PBF (%)	-0.23	0.76 (0.65-0.88)	**<0.001**	-0.11	0.90 (0.85-0.95)	**<0.001**
Grip strength (kg)	-0.16	0.85 (0.77-0.94)	**0.002**	-0.17	0.84 (0.77-0.92)	**<0.001**
Back muscle strength (kg)	-0.05	0.96 (0.93-0.98)	**0.002**	-0.05	0.95 (0.93-0.98)	**<0.001**

All variables (*p* < 0.01) that showed a certain degree of difference in univariate analysis were used as covariates. The dependent variable was presarcopenia. Covariates were body weight, PBF, NC by BIA, grip strength, and back muscle strength. Italicized values indicate significant difference. *β*: partial regression coefficient; CI: confidence intervals; NC: neck circumference; BIA: bioelectrical impedance analysis; PBF: percent body fat.

## Data Availability

The cohort data used to support the findings of this study are restricted by the Institutional Review Board of Nagoya University Graduate School of Medicine in order to protect the privacy of subjects in the Yakumo study.
